# Health Tract, No. 77

**Published:** 1865

**Authors:** 


### HEALTH TRACT, No. 77.

### HEALTH THEORIES.

Let the reader turn to the Health Tract, No. 76, and see how at one fell swoop the thousand and one stupid theories of crude minds, brass faces and wooden heads, are knocked into a tee-totally cocked-hat. You dish-water vegetarians, see what fools you have made of yourselves! Here is a Centenarian who never ate vegetables, but always did eat animal food, either milk or meat from the moment of his first squeak. And ye loud blatant cold-water soakers! Here is a jolly old chap of a hundred and five, who never saw a bath-tub; whose only shower-bath was a pouring rain in trying to keep some appointment; whose only *douche* was swimming some swollen "creek" in his endeavor to be up to time at some log-cabin meeting-house. What will that sapient Ohio Conference now do, who last year debated the resolution, if indeed they did not pass it, that any man who chewed tobacco should not be allowed to preach the Gospel? And you rampant ravers who have screamed yourselves blue in the face to prove that hog-meat is the cause of all the scrofula in the world, what think you of Father Cull's testimony, that almost the only meat he ever ate was "salt-meat," a word which out West is the synonym of ham and bacon. And in this connection, just turn to our article on "Pork as Food," for January 1859. We are not partial at all, and would "just as lief" give ourself a kick as any one else, if a "smile" can be got out of it. We have been proclaiming for years, that one of the best means of preserving health, especially "out West," was to take an early breakfast; and here is a man who has kept himself "out West" for a hundred years, has persisted in taking breakfast "late," and even in "fever'n-agy-Ingiany," has never had a "shake" in his life. In fact, this "old feller" seems to have lived "just a purpose" to demolish all speculation; he is the great theory-annihilator. Where is the grape-man, too, who had a large vineyard, and printed a book for gullible New-York, showing that the most certain way of living to a good old age, if not longer, was to eat grapes all the year round; to him, also, old Father Cull is a perfect Mississippi snag, for he couldn't abide any thing sour, nothing acid, not even that of fruit. Why, then, has the old man persisted in living all this time? that's what we would like to know, for we are very anxious to live a good while. We are like Paul, the spirit may be willing, but the flesh is weak; it will prefer the leeks and onions on this side Jordan. Come to think of it, we are rather more of the same mind with the little old fellow living on the Jersey Flats. The doctor told him his time had come; then they sent for the minister, who told him he ought to get ready, and more, he ought to be resigned and be willing. "Are you willing, Zechariah, to go to the other world?" "Oh! yes, I'll go," said Zec, with a faint and sighful voice; "but I had rather stay here where I'm better acquainted."

But perhaps it was the coffee that has kept our venerable friend alive. We suggest the formation of a society for promoting long life, whose whole system of by-laws, rules, regulations and constition shall be comprized in less than half a dozen words, easily remembered: Drink well-sweetened Coffee daily. Such a constitution is easily "expounded," can have but one interpretation, and is very readily carried into practice. Let a committee be appointed, with instructions to report progress on the first day of January, in the year of grace two thousand, and then have leave to report again, after a spell. The record which good old Father Cull has sent us of his life is very suggestive, as showing up a very common folly of weak minds, and there are multitudes of such in every department of human life, building theories of life and death, of human government and human conduct on single isolated facts. Facts are often falsehoods, because not taken with all their connections. Only whole facts are truths. It would be just as unfair to say that Father Cull has lived so long because he has always used tobacco, as it would be to aver that his great age is due to the fact that he never ate any thing sour, or that he never had any babies to keep him awake of nights, or gouge his eyes half out, or make digital explorations up his nostrils at day-light. The presumption is, that he lived so long because he had a good constitution to begin with; that the first half-century of his life was spent in industrious, useful and benign activities in the open air, combined with simple tastes, moderate appetites and as moderate an indulgence of the same. In short, he was a plain liver, a cheerful worker, and a good man from his youth up; and those of our readers who have an ambition to reach a patriarchal age, should, like Father Cull, live temperately and industriously, doing good always unto all men.

